# Effect of Antioxidant-Rich Moringa Leaves on Quality and Functional Properties of Strawberry Juice

**DOI:** 10.1155/2022/8563982

**Published:** 2022-09-30

**Authors:** Muhammad Adnan Arif, Muhammad Inam-ur-Raheem, Waseem Khalid, Clara Mariana Gonçalves Lima, Ravi Prakash Jha, Muhammad Zubair Khalid, Renata Ferreira Santana, Rohit Sharma, Abdulaziz Hassan Alhasaniah, Talha Bin Emran

**Affiliations:** ^1^National Institute of Food Science and Technology, University of Agricultural Faisalabad, Faisalabad, Pakistan; ^2^Department of Food Science, Government College University, Faisalabad, Pakistan; ^3^Department of Food Science, Federal University of Lavras, Lavras, Minas Gerais, Brazil; ^4^Department of Community Medicine, Institute of Medical Sciences, Banaras Hindu University, Varanasi, India; ^5^Department of Food Science, Southwestern Bahia State University, Itapetinga, Bahia, Brazil; ^6^Department of Rasa Shastra and Bhaishajya Kalpana, Faculty of Ayurveda, Institute of Medical Sciences, Banaras Hindu University, Varanasi 221005, Uttar Pradesh, India; ^7^Department of Clinical Laboratory Sciences, College of Applied Medical Sciences, Najran University, P.O. Box 1988, Najran, Saudi Arabia; ^8^Department of Pharmacy, BGC Trust University Bangladesh, Chittagong 4381, Bangladesh; ^9^Department of Pharmacy, Faculty of Allied Health Sciences, Daffodil International University, Dhaka 1207, Bangladesh

## Abstract

*Moringa oleifera* and strawberry are cultivated extensively worldwide and are divinely blessed with an enormous amount of nutritional and medicinal constituents, such as vitamin C, vitamin E, iron, potassium, and phenolic antioxidants that play a pivotal role in treating, confining, and preventing diabetes and many kinds of cancer. The focus of the study is to develop different samples of highly acceptable ready to serve (RTS) Moringa strawberry juice blend by underutilizing Moringa and strawberry juice in different proportions. *Moringa oleifera's* bitter taste and green color steeply limits its acceptability and counter this drawback utilized with strawberry juice. The physicochemical analysis of blended juice was performed to investigate the suitability and keeping quality of the juice mixture. The collected data signify that pH titratable acidity (TA) and total soluble solids (TSS) the slight modification after the inclusion of Moringa juice extract and throughout the storage. The Moringa treatment positively improved the total phenolic content (TPC), antioxidant, and vitamin C from 12 to 49.17 mg GAE/100g, 61.41 to 87.69%, and 64.03 to 86.65 mg/100 mL, respectively, but there was a slight decline in antioxidant quantity while stored under refrigerated conditions for one month. An assimilative trend was noticed in TPC and vitamin C, which collapsed from 49.17–36.32 mg GAE to 86.65–79.19 mg, respectively. In accordance with sensory analysis *T*_2_ (90% strawberry juice and 10% Moringa extract), the juice blend was rated best in context to flavor, color, and taste. This juice blend proved to be greatly effective especially for children suffering from malnutrition as well as women to counter with its appreciable number of nutritional constituents.

## 1. Introduction

Fruits are rich in nutrients but not acceptable due to high acidity, poor taste, and flavors. These fruits could be blended with other fruits to improve their acceptability and make use of available nutrients. The blending of fruit juice helps to improve nutritional status and reduce the cost of production by using cheaper fruits in the blends and also leads to new product development [1]. The blending of juices is helpful in maintaining the elevated levels of polyphenolic content and boosting the consumer preference for naturally sweet fruits with other polyphenolic-rich fruits [2, 3].

Many plant-based foods are not available throughout the year and their perishable nature makes them unfit for consumption due to such reasons as processing indispensable [4]. There are numerous technical applications that are administrated for the necessary modification of plant-based food in the context to improve its quality. The bioavailability of essential nutrients in plant-based foods is an aid to preserve the food from degradation. The blending is performed to achieve desired objectives, which is accomplished through the adulteration of one or more pure vegetable or fruit juices together for the value-added product. There are several reasons for forming the blend, such as to improve color, flavor, nutritional demand of pure fruits and vegetables, and also check-in production cost of the product.

*Moringa oleifera* (MO) belongs to the monogenic family Moringaceae, and has long been utilized as common human nutrition, medicinal, and industrial plant for centuries [5, 6]. It is an indigenous plant including 13 different species in Africa, America, and Asia and can be planted in both tropical and subtropical regions [7, 8]. The whole plant is blessed with numerous bioactive phytochemicals such as antioxidant (carotenoids), vitamins E (36.9 mg/100 g), C (271 mg/100 g), calcium (2003 mg), and iron (28.2 mg). These bioactive compounds are composed of several medicinal properties that are helpful in the treatment of diabetes, cancer, and many other diseases [7,9–12].

Strawberry fruits are famous among consumers due to their high bioactive compounds, appreciable sensory characteristics, and proven health promoting properties [13]. It is a perennial fruit that can be cultivated in both tropical and as well as in subtropical areas [14]. Normally, strawberries are red in color with 30–40 days maturity span [12] and also have very short storage life, almost 5–7 days at 0°C temperature with 95% of the relative humidity [15]. Strawberries possess elevated amounts of antioxidant property, which is especially due to the phenolic compounds and low glycemic index. These characteristics of the antioxidant and hypoglycemic effect are useful in curing and prevention against diabetes and other chronic ailments [16].

Apart from the above dividends of MO (*Moringa oleifera*) leaves, they also have several unpleasant aromatic compounds, such as saponins, those mainly responsible for their bitter taste, unacceptability, and dark green color [17]. It is detrimental that a beverage made by using Moringa leaf or its extract would need to have an acceptable flavor, taste, and color. They are very perishable in nature owing to their high moisture content and also have a short storage life.

Considering the above mentioned lapses with such elevated nutritional facts of *Moringa oleifera* and strawberry, the present study has been designed to introduce a nutritious and healthy juice blend prepared from Moringa and strawberry. Blending strawberry juice with Moringa leaf juice would provide a natural sweetening source to some extent in the beverage and increase the utilization of a highly perishable crop as well. The Moringa strawberry juice blend would have improved the color, flavor, and nutritional status and allot better appeal for the consumers with such benefits. The juice blend might be a handsome armor against several ailments, including diabetes, cancer, osteoporosis, heart problems, scurvy, and hemorrhages.

## 2. Materials and Methods

### 2.1. Raw Materials

Fully matured *Moringa oleifera* leaves were procured from the horticulture department of Agriculture University, Faisalabad, Pakistan, and fresh strawberries, chemicals, additional equipment, and packaging materials such as glass bottles and airtight glass jars were purchased from the local market in Faisalabad, Pakistan.

Ascorbic acid, metaphosphoric acid, 2,6 di-chlorophenolindophenol, and Folin-Ciocalteu's phenol reagent were procured from the National Institute of Food Science and Technology at the University of Agriculture, Faisalabad, Pakistan. The chemicals used in crude protein, standard gallic acid (GAE), deionized, sodium hydroxide (NaOH), methanol, and 2,2-diphenyl-1-picrylhydrazyl (DPPH), were taken from the Department of Food Science and Technology at Ayub Agriculture Research, Faisalabad, Pakistan.

#### 2.1.1. Moringa Leaf Juice Preparation

The fresh Moringa leaves were selected, sorted, and weighed after which they were washed twice with clean water. Then it was blanched for 2 min in boiling water and ground to form a paste. The prepared paste was mixed with 100 mL of water and then filtered through a muslin cloth to obtain filtered Moringa juice.

#### 2.1.2. Strawberry Juice Preparation

The fully matured and fresh strawberries were taken and the decayed parts were trimmed. After that, strawberries were washed thoroughly with tap water and then left to dry. After the strawberries were completely dry, a weighing balance was used for weight, and then the juice was prepared by adding water.

#### 2.1.3. Preparation of Drink

The blend of strawberry juice and Moringa juice was prepared at different treatments (100% strawberry juice, 95% strawberry juice + 5% Moringa juice, 90% strawberry juice + 10% Moringa juice, and 85% strawberry juice + 15% Moringa juice). The blend was mixed with a blender and then pasteurized at 80°C for 15 min. After the production of the Moringa-strawberry juice blend, the drink was packaged into labeling plastic containers and stored for further investigation in refrigerators at 4 ± 2°C.

### 2.2. Physicochemical Analysis

The total soluble solids (T.T.S.%), pH value, acidity, and ascorbic acid of different samples were evaluated in accordance with the method explained by AOAC [18]. Color evaluation (L^*∗*^, a^*∗*^, and b^*∗*^ values) via the methods elaborated by Meilgaard et al. [19].

### 2.3. Antioxidant Activity

#### 2.3.1. Sample Extraction

The extraction from the juice blend was made with the help of the protocol described by Arslan and Ozcan [20]. The exact amount of one gram extract was measured on a digital weighing balance and poured carefully into the test tubes already heaving 10 mL of 75% liquid methanol and mixed gently for one minute, then placed carefully in the centrifuge machine to separate the impurities of a sample at 20°C at 2500 RPM speed for 10 min. The mixture was then filtered, and the filtrated solution was further used for the evaluation of antioxidant activity and total phenolic compounds.

#### 2.3.2. Procedure

Applying DPPH (2-diphenyl-1-picrylhyDrazyl) and methanol extractions, the antioxidant activity was performed using the method described by Arsalan and Ozcan [20]. For each 5 mL of methanol extracted, a sample of juice blend of 0.1 mL was mixed with DPPH (2, 2-diphenyl-1-picrylhydrazyl) dye and the mixture was left in the darkness at ambient temperature for about 20 minutes as described in this method. The solution absorbance was measured with the assistance of a spectrophotometer at 517 nm wavelength after giving a rest at a 27°C temperature. By the following equation, the ability to radically scavenge DPPH was calculated:

(1) $$\begin{array}{c} \text{Antioxidant}\left(\%\right)=\frac{\text{OD Control}-\text{OD Sample}}{\text{OD Control}}. \end{array}$$

### 2.4. Determination of Total Phenolic Content

The Folin-Ciocalteau technique has estimated the complete phenolic content of the Moringa strawberry juice blend [20, 21]. The reaction blend included sample extracts of 100 *μ*L in a test tube and water distilled to 500 *μ*L in it. After adding 750 *μ*L of the Folin–Ciocalteu's reagent and leaving for the chemical response for the next 6 min, 6 g/100 mL of sodium carbonate was added to this reagent, then distilled water was added to the sample's final quantity of 3 mL. After 90 minutes at room temperature, the absorption at 760 nm was evaluated using a spectrophotometer by using conventional gallic acid to calculate the phenolic contents. (Genesys 10 UV, Thermo Scientific, Wisconsin, USA). The findings are displayed as mg of gallic acid per Gram of dry sample equivalent.

### 2.5. Total Plate Count

The total plate count of the Moringa strawberry juice blend was conducted through the method described by Lateef et al. [22]. For this evaluation, all apparatus used were subjected to sterilization in the hot air ovens at 171°C for continuous 30 minutes and the whole media was sterilized in the autoclave for the microbiological analysis. The TPCs were normalized with a logarithm base of 10. Duplicate analyses were conducted for each treatment.

(2) $$\begin{array}{c} \text{Total plate count}=\text{average bacterium number}\times \text{dilution factor}\times \text{volume factor}. \end{array}$$

### 2.6. Sensory Analysis

The 9-point hedonic scale was used for the sensory analysis of all the treatments of the Moringa strawberry juice blend, as described by [19]. The Moringa strawberry juice blend's sensory analysis was performed on the basis of its taste, flavor, color, and texture. The sensory assessment of the parameters was carried out on the same day at environmental temperature and the same procedure was repeated. The panelist allotted the score according to the scale at the National Institute of Food Science and Technology (NIFSAT), University of Agriculture, Faisalabad, Pakistan, for one month on a weekly basis.

### 2.7. Statistical Analysis

The gathered information was used to show the optimization by implementing a completely randomized design for each physicochemical and sensory parameter. The levels in the sensory attributes (color, flavor taste, and texture) and physicochemical parameters (pH, acidity, ascorbic acid, and TPC) of the significant differences (*p* ≤ 0.05) were determined by applying two-factor CRD by following the principles described by Steel [23].

## 3. Results and Discussion

### 3.1. Physicochemical Analysis

#### 3.1.1. pH

The results regarding the mean value of pH are shown in Table 1, where the calculated pH lies between 4.19 and 4.82 among treatments during the entire storage period. The treatment *T*_3_ indicated the highest pH values during the storage period, while control *T*_0_ showed the lowest pH values during the storage time. The pH was significantly different between storage days and a decreasing trend had been observed. In the different treatments, the pH was significantly altered and was increased between *T*_0_ and *T*_3_. In *T*3 and *T*0, the maximum and minimum pH were observed, respectively.

The study observed the decline in the pH of treated samples during the period of storage. The decreasing trend of pH was observed up to 28 days of refrigerated storage. The results regarding pH are also similar to the study conducted by Hashemi. et al. [24], who used *Moringa oleifera* leaves extract for extending the shelf life and also improving the quality of fresh sweet orange juice.

#### 3.1.2. Acidity (%)

The mean values of acidity are shown among various storage intervals and treatments in Table 1, where the acidity was ranged from 0.03 to 0.36 on different treatments during the whole storage period. There was a gradual decrease in the acidity value as the *Moringa oleifera* leaves extract proportion increased in the blend. The treatment *T*_3_ (15 mL of Moringa and 85 mL of strawberry) indicated the lowest acidity value of 0.03 during the storage at 0 days, while the control *T*_0_ (untreated strawberry juice) showed the highest acidity percentage of 0.36 during the storage time of 28 days.

Similar findings were confirmed during the 20 days of storage by Baljeet et al. [25] in whey-based pineapple and bitter gourd. Din et al. [26] noted an increase in acidity percentage in all treatments during storage of functional beverages prepared from the bitter gourd juice. Raj et al. [27] also noticed an increase in acidity during storage time in apple juice and sand pear's all the formulations. This increase in acidity during the Moringa strawberry drink might be the result of lactose conversion into lactic acid and also due to the formation of other organic acids in the presence of ascorbic acid.

#### 3.1.3. Total Soluble Solids

The findings for mean values show that the total soluble solids were compared between different storage intervals and treatments in Table 1, where the calculated total soluble solids lie between 1.11 and 1.47 among treatments during the entire storage period. The treatment *T*_3_ (15 mL of Moringa and 85 mL of strawberry) indicated the highest total soluble solids value of 1.47 during the storage at 0 days, while control *T*_0_ (untreated) showed the lowest total soluble solids value of 1.11 for the 28 days of storage time. The total soluble solids showed a highly significant among storage days and exhibited a decreasing trend. A different scenario was observed among treatments in which total soluble solids changed significantly, and the total soluble solids trend in *T*_0_ to *T*_3_ was increasing.

The observed results regarding total soluble solids content exhibited closeness to the study conducted by Akande and Ojekemi [28], who blended the watermelon and pineapple juice blend. Probably owing to enzymes responsible for the growing trends in total soluble solids or the reason for the inversion of sucrose could be a high temperature, which might increase the value of total soluble solids.

### 3.2. Ascorbic Acid (mg/100 mL)

The results indicate the comparison of mean values of ascorbic acid among various storage intervals and treatments in Table 2, where the calculated total soluble solids lie between 56.46 and 86.65 among treatments during the whole storage period. The treatment *T*_3_ (15 mL of Moringa and 85 mL of strawberry) reported the highest ascorbic acid value (86.65 mg/100 mL) during the storage at 0 days, while control *T*_0_ (untreated strawberry juice) showed the lowest ascorbic acid value (56.46 mg/100 mL) for twenty-eight (28) days in storage. The ascorbic acid showed a significant decreasing trend among storage days.

Islam et al. [29] investigated a declining trend of ascorbic acid in many blends of orange and pineapple juice that were for 35 days at refrigeration temperature. The highest decrease in ascorbic acid (37.1–12.6 mg/100 mL) was cited in a sample (50 : 50) ratio of orange and pineapple juice. Due to variables like metal contamination, oxidative enzymes, processing methods, oxygen presence in the head area, and the decline in ascorbic acid trend, it was conceivable during storage due to storage temperatures.

### 3.3. Total Phenolic Contents

**Figure 1:**
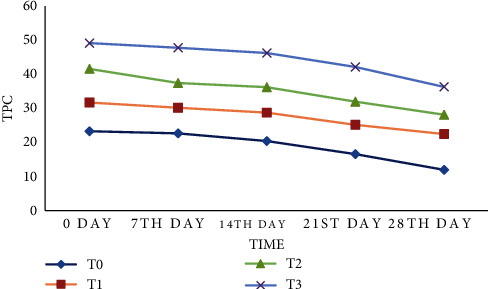
Effect of treatment and storage on phenolic content of Moringa and strawberry juice.

The results indicate the comparison of mean values of TPC among various storage intervals and treatments in Figure 1. The collected data showed that Moringa and strawberry juice treatments influence the total phenolic content of the juice blend (GAE/100 g) significantly. The calculated total phenolic contents lie between 12.0 and 49.17 among the treatments during the entire storage period. The highest total phenolic contents (49.17 mg GAE/100g) were observed in treatment *T*_3_ (15 mL Moringa and 85 mL strawberry) at 0 days, while control *T*_0_ (untreated) showed the lowest total phenolic content value (12 mg GAE/100g) at the time of 28 days.

The observed results regarding total phenolic contents exhibit closeness to the study conducted by Ali et al. [30]; who developed a guava whey beverage fortified with *Moringa oleifera* leaves extract. Similarly, a declining trend of total phenolics was reported by Mena et al. [31] who investigated the combinatory effect of thermal treatment and blending on the quality of pomegranate juices. The declining trend of total phenolic content was probably caused by inhibiting the transformation of proteins to sugars and salts through enzymes by Moringa and strawberries during storage.

### 3.4. Antioxidant (%)

Table 3 shows the assimilation results among various treatments and the storage time in terms of mean score values. The calculated data on antioxidants lie between 56.27 and 87.69 for different parameters. The treatment *T*_3_ (15 mL of Moringa and 85 mL of strawberry) indicated the highest antioxidant quantity of 1.47 during the storage at 0 days, while control *T*_0_ (untreated) during the 28-day storage period showed the smallest antioxidant value of 56.27. The antioxidants showed a high significance among storage days and exhibited a decreasing trend.

The results observed in relation to antioxidant content were relative size to the study of a mixture of watermelon and apple juice developed by Akande and Ojekemi [28]. Deka and Sethi [32], for their anola lime/pineapple mango juice mixtures, have observed the boost in the RTS-spiced drink's antioxidants stored at low temperatures. The growing trend of antioxidants likely owing to enzymatic reactions or elevated temperature may be the reason behind the sucrose conversion, which may result in the antioxidant value being increased.

### 3.5. Color

The colorimeter was used to determine the color parameters of the Moringa strawberry juice blend at each time interval of the storage period. The color parameters L^*∗*^ indicated the lightness while a^*∗*^ and b^*∗*^ were chromaticity parameters, which indicated the red-green and blue-yellow values, respectively. A higher value of the L^*∗*^ indicates maximum brightness of the samples.

#### 3.5.1. L^*∗*^ (Lightness) Value

The L^*∗*^ value of all treatments is shown in Table 4, where the value ranges from 11.18 to 26.64 across the entire storage period. The significant impact of days on the L^*∗*^ value of Moringa strawberry blends was observed, and the value shows an increasing trend with the storage period. The impact of the treatment on the L^*∗*^ value was not significant. At 0 days, the L^*∗*^ value showed a decreasing trend from *T*_0_ and *T*_3,_ and the same scenario was observed at 28 days. The peak value of L^*∗*^ was 26,64 on T0 at 0 day, whereas the minimum value of L^*∗*^ was 11,18, on T3 at 28 days.

The L^*∗*^ results were similar to Hashemi et al. [24], who used *Moringa oleifera* leaves extract to extend the storage life and improve the freshly sweet orange flavorful quality, as well. The results were similar to those of Choi et al. [33], who analyzed the effect of ascorbic acid retention on the color of juice and the pigment stability of orange juice (Citrus sinensis) under refrigeration. The change in the amount of the L^*∗*^ value of the prepared Moringa strawberry juice mixture might be due to the storage period's function.

#### 3.5.2. a^*∗*^ (Red-Green) Value

The results of the a^*∗*^ value are given in Table 4 where a^*∗*^ value ranged from 0.88 to 1.24 during the entire storage period. The value of a^*∗*^ decreases from *T*_0_ to *T*_3_ and each storage interval noted the highly significant impact of these medicines on a^*∗*^ value. The trend of a^*∗*^ value had been increasing between 0 and 7 storage days, and then 7 to 14 days observed an increasing trend after that was again visible in Table 4. After that, the decreasing trend from 14 to 28 days' of refrigerator storage and the impact of days on a^*∗*^ value were observed to be nonsignificant. The minimum value of a^*∗*^ appeared to be at 7 days in *T*_3_ and the maximum value of a^*∗*^ was observed in *T*_0_ at 0 days of storage. At both 0 and 28 days of storage, all the treatments presented minimum and maximum values.

The results of a^*∗*^ value were analogous to the findings of Hashemi et al. [24], who used *Moringa oleifera* leaves extract to extend the shelf life and also improve the quality of fresh sweet orange juice. Results were also compared to Choi et al. [33], who evaluated the impact of juice color and pigment stability in orange juice (Citrus Sinensis) while stored and chilled, on ascorbic acid retention. The change in the quantity of a^*∗*^ value of the ready-to-serve Moringa strawberry juice blend might be due to the function of storage time.

#### 3.5.3. b^*∗*^ Value (Color)

The results of b^*∗*^ value are given in Table 4 where b^*∗*^ value ranged from 0.88 to 1.24 during the entire storage period. The value of b^*∗*^ decreases from *T*_0_ to *T*_3_ and the highly significant effect of these treatments on b^*∗*^ value at each storage interval has been observed. An increasing trend of b^*∗*^ value was observed from 0 to 7 days of storage and then 7 to 14 days observed an increasing trend after that again visible in Table 4, a decreasing trend from 14 to 28 days' refrigerator storage, and the impact of days on b^*∗*^ value was observed to be nonsignificant. The minimum value of b^*∗*^ appeared to be at 7 days in *T*_3_ and the maximum value of b^*∗*^ was observed in *T*_0_ at 0 days of storage.

The results of b^*∗*^ value were analogous to the findings of Hashemi et al. [24], who used *Moringa oleifera* leaves extract to extend the shelf life and also improve the quality of fresh sweet orange juice. The results acquired are also comparable to the research conducted by Choi et al. [33]; the effect on juice color and pigment stabilization in blood orange juice (*Citrus sinensis*) has been investigated while the storage conditions for cooling has been assessed. The change in the quantity of b^*∗*^ value of the ready-to-serve Moringa strawberry juice blend might be due to the function of storage time.

### 3.6. Microbiological Analysis

#### 3.6.1. Total Plate Count

The results of means regarding total plate count are given in Table 5, in which the value ranged from 4.71 to 6.55 log_10_ CFU/g. The treatments caused a significant decrease in the value of total plate count, whereas the storage days' impact was highly significant on the coliform count, and the value showed an increasing trend from 0 to 28 days of storage. The interaction of both treatments and days produced a significant impact on the plate count of the fish patties. The total coliform count of control (uncoated) treatment *T*_0_ was significantly higher than the coated treatments *T*_1_–*T*_4_. The maximum and minimum values of the total coliform count were shown by *T*_0_ at 28 days and *T*_4_ at 0 days of storage.

The findings of the current research are in accordance with the results of Hashemi et al. [24] as the investigated microbiological impact of *Moringa oleifera* leave extract (M.O.L.E) in guava whey juice during the storage time at 4°C±1 till one month. The outcomes illustrated that the control A (70% orange juice + 20% water + 10% ginger) exhibited the maximum total bacterial counts of (21 × 10^3^) up to one month, but sample B (80% whey protein +20% guava +1.5% M.O.L.E) and C (80% whey protein +20% guava +2% M.O.L.E) did not show any bacterial count even after 2 months.

### 3.7. Sensory Analysis

#### 3.7.1. Color

**Figure 2:**
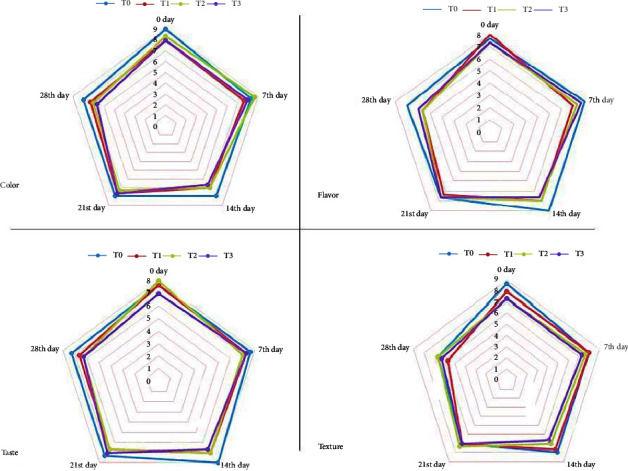
Sensory analysis of Moringa strawberry blend during storage.

The mean scores of appearances of stored Moringa strawberry blend samples are given in Figure 2, in which the score ranged from 6.67 to 9. The storage days impacted highly significantly on the score, and the mean score showed in Figure 2 a decreasing trend from 0 to 28 days of storage. The interaction in both the treatment and days did not produce any significant impact on the color of the Moringa strawberry blends. The color score of the control untreated sample *T*_0_ was higher than the extract containing treatments *T*_1_–*T*_3_. The maximum and minimum values of the score were shown by *T*_0_ at 0 days and *T*_3_ at 28 days.

The decline in color through the storage intervals was investigated by Safdar *et al.* [34] in guava leather drinks stored in different packaging materials. A gradual decreasing trend in color for all the packaging materials was witnessed, as storage time increased. Kausar et al. [35] investigation in agreement with the findings of the decreasing color rating during a storage period of 4 months in a cucumber and muskmelon juice blend.

#### 3.7.2. Flavor

The mean scores of the flavors of stored Moringa strawberry blend samples are given in Figure 2, in which the score was ranged from 5.67 to 8. The treatments caused a highly significant decrease in the mean score of flavors, whereas the storage days also impacted highly significantly on the score, and the mean score showed in Figure 2 a decreasing trend from 0 to 28 days of storage. The interaction in both of the treatment and days did not show any significant impact on the color of the Moringa strawberry juice blends. The flavor score of control untreated sample *T*_0_ was higher than the extract containing treatments T_1_-T_3_. The maximum and minimum values of the score were shown by *T*_0_ at 0 days and *T*_3_ at 28 days of refrigeration storage.

The declining trend was analogously observed in a study conducted by Sangma et al. [36], who prepared RTS drinks via blending different ratios of Aloe vera ginger and sweet lime. They observed a gradual decrease in the flavor of the beverage up to 8 weeks of storage. They investigated the maximum decline range noticed in *T*_2_ (8.55–5.70) and followed by T3 (8.85–6.10) in various ratios of *Aloe vera*: ginger, and sweet lime: amla 40 : 5:40 : 15) and (60 : 5:20 : 15) respectively.

#### 3.7.3. Taste

The mean scores of tastes of stored juice samples are given in Figure 2, in which the score ranged from 6.33 to 8. The treatments caused a significant decrease in the score of taste, whereas the storage days significantly decreased the taste. *T*_2_ at 0 days, *T*_0_ at 14 days, and *T*_3_ at 28 storage days showed the highest and minimum rank values. The highest mean score in *T*_0_ when there was no Moringa extract, and as the Moringa extract increased, the mean score of the juice blend decreased, as shown in Figure 2.

The orange and carrot juice blend findings were in agreement as described by Raj et al. [27]. They studied the storage investigation in several blends of sand pear and apple juice. They noticed the maximum decline in the taste score of the drink in *T*_3_ (8.6–8.3) and then followed as *T*_2_ (8.1–7.9) in sand pear-apple juice blending ratios (80 : 20) and (70 : 30), respectively. Kausar et al. [35] manufactured a functional beverage via the blending of cucumber and muskmelon in different formulations and reported similar findings as a decrease in the drink's taste rating during storage intervals.

#### 3.7.4. Texture

The mean scores of the textures of stored Moringa strawberry blend samples are given in Figure 2, in which the score ranged from 5.67 to 8.67. The treatments caused a nonsignificant decrease in the texture score, whereas the storage days significantly decreased the texture. The texture score of control treatment *T*_0_ was higher than the *Moringa oleifera* leaves extract treated blends in treatments from *T*_1_ and *T*_3_. The maximum and minimum values of the score were shown by *T*_0_ at 0 days and *T*_1_ after 28 days of storage. The highest mean score in T0 when there is no Moringa extract and Moringa extract increased the mean score of the juice blend decreased, as shown in Figure 2.

The decline in texture through the storage intervals was investigated by Safdar et al. [34] in guava leather drinks stored in different packaging materials. They witnessed a gradual decreasing trend in texture for all the packaging materials as storage time increased. Kausar et al. [35] investigated in agreement with the findings of decreasing texture rating during a storage period of 4 months in a cucumber and muskmelon juice blend.

## 4. Conclusion

To sum up overall, as previously mentioned, Moringa is infamous owing to its color and bad taste for this reason, it is accompanied by strawberry juice to enhance the utilization of this miracle plant. Studies indicate that up to 10% is quite acceptable as beyond this amount negatively influences the sensory characteristics of the product although it boosts nutritional compounds. The juice blend is unbelievably astonishing to prevent malnutrition in all ages and genders as Moringa ridiculously improves nutritional constituents.

## Figures and Tables

**Table 1:** Changes in pH, acidity, and TSS of Moringa strawberry blend during storage.

Treatments	pH					Acidity					Total soluble solids				
	0 day	7 days	14 days	21 days	28 days	0 day	7 days	14 days	21 days	28 days	0 day	7 days	14 days	21 days	28 days
*T*_o_	4.35 ± 0.03^au^	4.34 ± 0.02^au^	4.28 ± 0.01^av^	4.23 ± 0.02^v^	4.19 ± 0.01^x^	0.19 ± 0.10^au^	0.27 ± 0.20^av^	0.28 ± 0.05^av^	0.30 ± 0.07^av^	0.36 ± 0.17^ay^	1.31 ± 0.01^au^	1.30 ± 0.03^au^	1.21 ± 0.05^av^	1.20 ± 0.07^av^	1.11 ± 0.02^ax^
*T*_1_	4.66 ± 0.07^bu^	4.65 ± 0.15^buw^	4.60 ± 0.06^buw^	4.55 ± 0.0^1bvw^	4.46 ± 0.07^bv^	0.12 ± 1.07^bu^	0.20 ± 1.15^av^	0.22 ± 0.72^bvx^	0.25 ± 0.09^awxy^	0.29 ± 0.97^by^	1.37 ± 0.07^bu^	1.35 ± 0.15^bu^	1.32 ± 0.72^bv^	1.27 ± 0.09^bw^	1.19 ± 0.06^bx^
*T*_2_	4.80 ± 0.08^cu^	4.75 ± 0.02^cuv^	4.72 ± 0.87^cvw^	4.66 ± 0.20^bew^	4.59 ± 0.03^cx^	0.07 ± 1.87^cu^	0.09 ± 1.02^buv^	0.12 ± 0.87^cv^	0.15 ± 0.29^bvx^	0.19 ± 1.71^cx^	1.42 ± 0.05^cu^	1.40 ± 0.02^buw^	1.33 ± 0.06^bv^	1.32 ± 0.09^bdvw^	1.23 ± 0.71^cx^
*T*_3_	4.82 ± 0.03^uc^	4.80 ± 0.02^duv^	4.75 ± 0.05^cv^	4.75 ± 0.05^ev^	4.62 ± 0.06^cx^	0.03 ± 0.91^cu^	0.10 ± 0.87^buv^	0.12 ± 0.65^cv^	0.16 ± 0.98^cw^	0.20 ± 1.10^cw^	1.47 ± 0.91^du^	1.45 ± 0.32^du^	1.40 ± 0.05^dv^	1.34 ± 0.08^dw^	1.29 ± 0.10^dw^

*T *_o_ = 100% strawberry juice; *T*_1_ = 95% strawberry juice +05% Moringa extract; *T*_2_ = 90% strawberry juice +10% Moringa extract; *T*_3_ = 85% strawberry juice +15% Moringa extract; The values are mean ± *SD* of three independent determinations. Means carrying different letters in a column differed significantly (*p* < 0.05); a-e values with different letters within a column are significantly different (*p* < 0.05); u-y values with different letter within a row are significantly different (*p* < 0.05).

**Table 2:** Vitamin C of Moringa strawberry blend during storage.

Treatments	Vitamin C				
	0 day	7 days	14 days	21 days	28 days
*T*_o_	64.03 ± 0.10^au^	61.29 ± 0.20^av^	60.12 ± 0.05^aw^	58.74 ± 0.07^ax^	56.46 ± 0.17^ay^
*T*_1_	71.55 ± 1.07^bu^	69.34 ± 1.15^bv^	67.58 ± 0.72^bw^	65.18 ± 0.09^bx^	63.74 ± 0.97^by^
*T*_2_	79.11 ± 1.87^cu^	77.05 ± 1.02^cv^	75.88 ± 0.87^cv^	73.20 ± 0.29^cw^	71.81 ± 1.71^cx^
*T*_3_	86.65 ± 0.91^du^	84.77 ± 0.87^dv^	82.59 ± 0.65^dw^	81.04 ± 0.98^dx^	79.19 ± 1.10^dy^
Mean	75.34	73.11	71.54	69.54	67.80

*T *_o_ = 100% strawberry juice; *T*_1_ = 95% strawberry juice +05% Moringa extract; *T*_2_ = 90% strawberry juice +10% Moringa extract; *T*_3_ = 85% strawberry juice +15% Moringa extract; The values are mean ± *SD* of three independent determinations. Means carrying different letters in a column differed significantly (*p* < 0.05); a-e values with different letters within a column are significantly different (*p* < 0.05); u-y values with different letters within a row are significantly different (*p* < 0.05).

**Table 3:** Antioxidant of Moringa strawberry blend during storage.

Treatments	Antioxidant				
	0 day	7 days	14 days	21 days	28 days
*T*_o_	61.41 ± 0.97^au^	57.47 ± 0.98^au^	59.00 ± 1.00^av^	59.00 ± 0.12^aw^	56.27 ± 1.01^ax^
*T*_1_	71.19 ± 0.07^bu^	66.75 ± 0.98^bv^	67.75 ± 0.61^bv^	68.07 ± 0.43^bx^	64.95 ± 0.98^by^
*T*_2_	79.090.65^cu^	74.78 ± 0.76^cu^	77.28 ± 0.33^cv^	77.73 ± 0.23^cw^	73.66 ± 0.76^cx^
*T*_3_	87.69 ± 0.23^du^	83.47 ± 0.54^dv^	84.26 ± 0.81^dv^	86.28 ± 0.43^dx^	81.56 ± 1.09^dy^
Mean	74.85	70.62	72.07	72.90	69.11

*T *_o_ = 100% strawberry juice; *T*_1_ = 95% strawberry juice +05% Moringa extract; *T*_2_ = 90% strawberry juice +10% Moringa extract; *T*_3_ = 85% strawberry juice +15% Moringa extract; The values are mean ± *SD* of three independent determinations. Means carrying different letters in a column differed significantly (*p* < 0.05); a-e values with different letters within a column are significantly different (*p* < 0.05); u-y values with different letters within a row are significantly different (*p* < 0.05).

**Table 4:** Changes in color value of Moringa strawberry blends during storage.

Treatments	L^*∗*^					a^*∗*^					b^*∗*^				
	0 day	7 days	14 days	21 days	28 days	0 day	7 days	14 days	21 days	28 days	0 day	7 days	14 days	21 days	28 days
*T*_o_	26.64 ± 0.90^abu^	23.11 ± 0.60^av^	21.24 ± 0.67^aw^	18.51 ± 0.09^ax^	15.29 ± 0.25^ay^	1.19 ± 0.20^au^	1.14 ± 0.10^av^	1.17 ± 0.04^au^	1.16 ± 0.06^aw^	1.12 ± 0.18^auw^	0.56 ± 0.30^au^	0.60 ± 0.60^av^	0.65 ± 0.05^aw^	0.70 ± 0.09^aw^	0.77 ± 0.15^ay^
*T*_1_	22.38 ± 0.07^au^	19.43 ± 0.55^buv^	18.84 ± 0.54^bv^	15.23 ± 0.86^bw^	11.88 ± 0.45^bx^	1.13 ± 1.37^bu^	1.06 ± 1.26^bv^	1.13 ± 0.64^auv^	1.10 ± 0.06^bw^	1.05 ± 0.77^bw^	0.46 ± 0.06^bu^	0.37 ± 0.65^bv^	0.34 ± 0.02^bvy^	0.45 ± 0.19^bx^	0.31 ± 0.87^bxy^
*T*_2_	23.51 ± 0.67^bu^	20.74 ± 0.42^cu^	18.91 ± 0.43^cv^	15.66 ± 0.45^cw^	12.38 ± 0.61^cx^	1.24 ± 1.57^cux^	1.17 ± 1.05^cv^	1.23 ± 0.65^buv^	1.20 ± 0.12^cx^	1.17 ± 1.51^aw^	0.40 ± 0.57^au^	0.30 ± 0.72^bv^	0.28 ± 0.57^bw^	0.35 ± 0.39^cw^	0.26 ± 0.71^bx^
*T*_3_	23.46 ± 0.81^abu^	19.96 ± 0.65^du^	17.09 ± 0.43^dv^	14.88 ± 0.32^dw^	11.39 ± 0.13^dx^	0.95 ± 0.61^du^	0.88 ± 0.58^dvy^	0.91 ± 0.43^cuy^	0.90 ± 0.78^dx^	0.89 ± 1.20^cx^	0.29 ± 0.81^du^	0.22 ± 0.75^cuv^	0.21 ± 0.61^dvx^	0.28 ± 0.21^bcw^	0.17 ± 0.13^cwx^

*T *_o_ = 100% strawberry juice; *T*_1_ = 95% strawberry juice +05% Moringa extract; *T*_2_ = 90% strawberry juice +10% Moringa extract; *T*_3_ = 85% strawberry juice +15% Moringa extract; The values are mean ± *SD* of three independent determinations. Means carrying different letters in a column differed significantly (*p* < 0.05); a-e values with different letters within a column are significantly different (*p* < 0.05); u-y values with different letters within a row are significantly different (*p* < 0.05).

**Table 5:** Total coliform bacterial count (log_10_ CFU/g) of Moringa strawberry blend during storage.

Treatments	Total coliform bacterial count (log_10_ CFU/g)				
	0 day	7 days	14 days	21 days	28 days
*T*_o_	4.73 ± 0.05^au^	5.36 ± 0.10^abv^	6.45 ± 0.50^abv^	6.61 ± 0.30^abv^	6.81 ± 0.65^abv^
*T*_1_	4.71 ± 0.07^au^	5.55 ± 0.20^bv^	6.45 ± 0.07^bcw^	6.57 ± 0.08^ax^	6.59 ± 0.06^bcx^
*T*_2_	4.72 ± 0.04^au^	5.31 ± 0.16^av^	6.22 ± 0.04^aw^	6.40 ± 0.09^bx^	6.49 ± 0.09^ay^
*T*_3_	4.71 ± 0.09^au^	6.13 ± 0.04^cvx^	6.14 ± 0.50^acvy^	6.28 ± 0.10^ay^	6.39 ± 0.54^acxy^

*T *_o_ = 100% strawberry juice; *T*_1_ = 95% strawberry juice + 05% Moringa extract; *T*_2_ = 90% strawberry juice + 10% Moringa extract; *T*_3_ = 85% strawberry juice + 15% Moringa extract; The values are mean ± *SD* of three independent determinations. Means carrying different letters in a column differed significantly (*p* < 0.05); a-e values with different letter within a column are significantly different (*p* < 0.05); u-y values with different letter within a row are significantly different *p* < 0.05).

## Data Availability

All data used to support the findings of this study are included within the article.
